# Automated Deep Learning Phenotyping of Tricuspid Regurgitation in Echocardiography

**DOI:** 10.1001/jamacardio.2025.0498

**Published:** 2025-04-16

**Authors:** Amey Vrudhula, Milos Vukadinovic, Christiane Haeffele, Alan C. Kwan, Daniel Berman, David Liang, Robert Siegel, Susan Cheng, David Ouyang

**Affiliations:** 1 Smidt Heart Institute, Department of Cardiology, Cedars-Sinai Medical Center, Los Angeles, California; 2 Icahn School of Medicine at Mt Sinai, New York, New York; 3 Department of Bioengineering, University of California, Los Angeles; 4 Division of Cardiology, Department of Medicine, Stanford University, Palo Alto, California; 5 Division of Artificial Intelligence in Medicine, Cedars-Sinai Medical Center, Los Angeles, California

## Abstract

**Importance:**

Accurate assessment of tricuspid regurgitation (TR) is necessary for identification and risk stratification.

**Objective:**

To design a deep learning computer vision workflow for identifying color Doppler echocardiogram videos and characterizing TR severity.

**Design, Setting, and Participants:**

An automated deep learning workflow was developed using 47 312 studies (2 079 898 videos) from Cedars-Sinai Medical Center (CSMC) between 2011 and 2021. Data analysis was performed in 2024. The pipeline was tested on a temporally distinct test set of 2462 studies (108 138 videos) obtained in 2022 at CSMC and a geographically distinct cohort of 5549 studies (278 377 videos) from Stanford Healthcare (SHC). Training and validation cohorts contained data from 31 708 patients at CSMC receiving care between 2011 and 2021. Patients were chosen for parity across TR severity classes, with no exclusion criteria based on other clinical or demographic characteristics. The 2022 CSMC test cohort and SHC test cohorts contained studies from 2170 patients and 5014 patients, respectively.

**Exposure:**

Deep learning computer vision model.

**Main Outcomes and Measures:**

The main outcomes were area under the receiver operating characteristic curve (AUC), sensitivity, and specificity in identifying apical 4-chamber (A4C) videos with color Doppler across the tricuspid valve and AUC in identifying studies with moderate to severe or severe TR.

**Results:**

In the CSMC test dataset, the view classifier demonstrated an AUC of 1.000 (95% CI, 0.999-1.000) and identified at least 1 A4C video with color Doppler across the tricuspid valve in 2410 of 2462 studies with a sensitivity of 0.975 (95% CI, 0.968-0.982) and a specificity of 1.000 (95% CI, 1.000-1.000). In the CSMC test cohort, moderate or severe TR was detected with an AUC of 0.928 (95% CI, 0.913-0.943), and severe TR was detected with an AUC of 0.956 (95% CI, 0.940-0.969). In the SHC cohort, the view classifier correctly identified at least 1 TR color Doppler video in 5268 of the 5549 studies, resulting in an AUC of 0.999 (95% CI, 0.998-0.999), a sensitivity of 0.949 (95% CI, 0.944-0.955), and a specificity of 0.999 (95% CI, 0.999-0.999). The artificial intelligence model detected moderate or severe TR with an AUC of 0.951 (95% CI, 0.938-0.962) and severe TR with an AUC of 0.980 (95% CI, 0.966-0.988).

**Conclusions and Relevance:**

In this study, an automated pipeline was developed to identify clinically significant TR with excellent performance. With open-source code and weights, this project can serve as the foundation for future prospective evaluation of artificial intelligence–assisted workflows in echocardiography.

## Key Points

**Question:**

Can an automated deep learning workflow accurately assess presence and severity of tricuspid regurgitation (TR) from full transthoracic echocardiography (TTE) studies at a large scale?

**Findings:**

In geographically and temporally distinct test cohorts, the deep learning workflow showed strong and consistent performance in identifying apical 4-chamber videos with color Doppler across the tricuspid valve (area under the receiver operating characteristic curve [AUC], ≥0.999) and in detecting studies with clinically significant TR (AUC, ≥0.928).

**Meaning:**

In this study, an end-to-end deep learning pipeline automatically identified clinically significant TR from thousands of full TTE studies with excellent performance.

## Teaser

This study describes the development and testing of a deep learning computer vision workflow for identifying color Doppler echocardiogram videos and characterizing tricuspid regurgitation presence and severity using cohorts from 2 sites.

## Introduction

Accurate and reliable assessment of tricuspid regurgitation (TR) severity remains an ongoing challenge. Once considered a benign consequence of coexisting heart disease, TR has received more recent recognition as an independent risk factor for morbidity and mortality.^1,2,3^ While recent findings highlight the need for earlier diagnosis and monitoring, TR can often be asymptomatic and present without auscultation findings.^4^ With high temporal resolution, transthoracic echocardiography (TTE) is the most common test of choice for characterizing TR; however, accurate diagnosis requires expert assessment, as there is significant intraobserver variability.^5,6^ As new therapeutic options for treating TR, like percutaneous repair, emerge,^7,8^ early and accurate diagnosis has become more important.

Recent advances in computer vision and artificial intelligence (AI) have enabled precision phenotyping of structure and function in cardiac ultrasound.^9^ AI applied to echocardiography can precisely estimate wall thickness^10^ and assess mitral regurgitation severity^11^ and left ventricular ejection fraction (LVEF),^12,13^ as well as detect cardiac amyloidosis,^10,14^ hypertrophic cardiomyopathy,^15^ and diastolic dysfunction.^16^ Application of deep learning for TR has lagged behind, with most machine learning approaches using structured tabular data to characterize and prognosticate TR rather than evaluating the underlying images themselves.^17,18,19,20^ AI guidance has been developed for both image acquisition and interpretation,^12,21^ and given the increasing prevalence of TR in an aging population with comorbid heart failure, AI could aid in TR screening and surveillance.^22,23,24,25,26^

**Figure 1.**
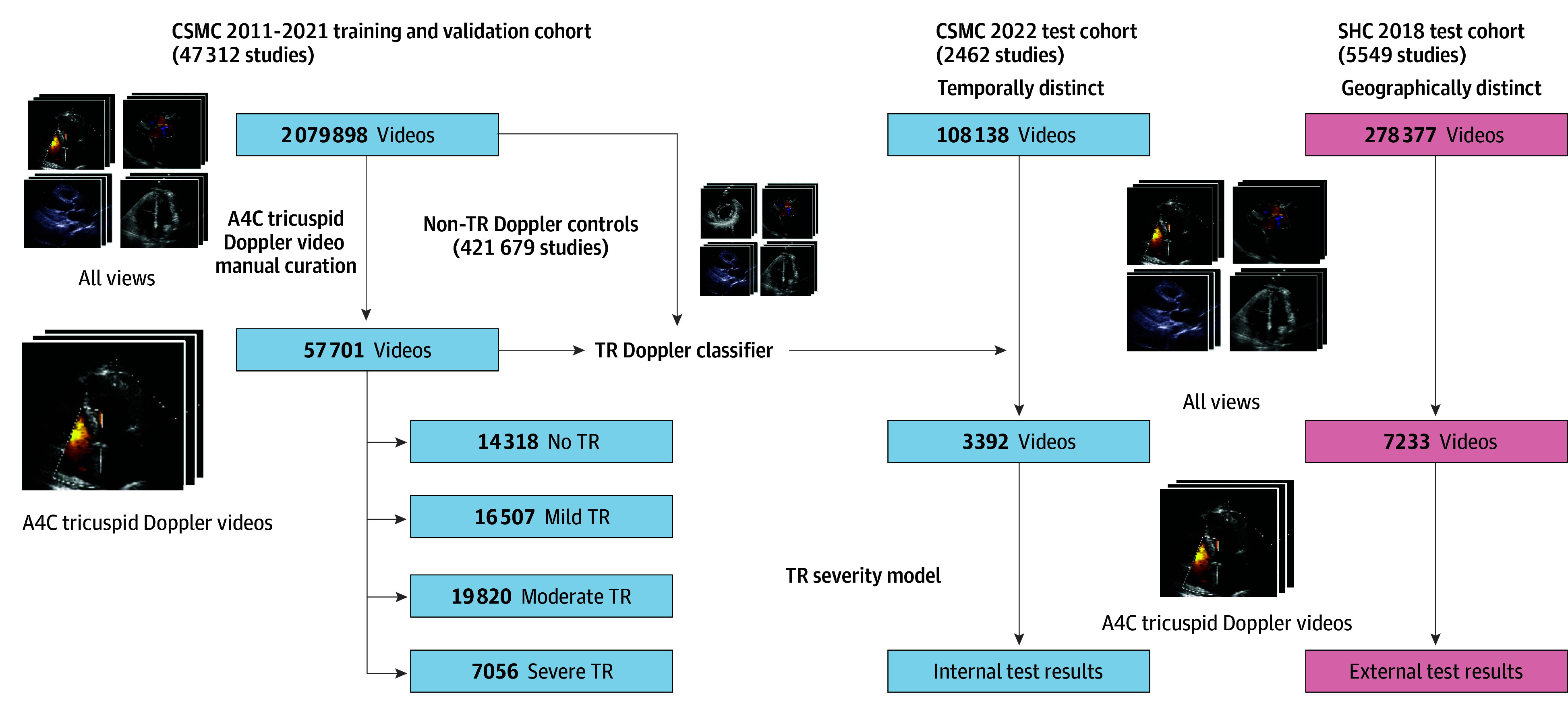
Dataset Isolation and Automated Tricuspid Regurgitation (TR) Detection Pipeline Development A total of 57 701 apical 4-chamber (A4C) videos were manually curated from 47 312 studies with varying TR severity and used to train deep learning models for view classification and TR severity stratification. A pipeline consisting of these models was then benchmarked on a temporally distinct cohort of 2462 studies from Cedars-Sinai Medical Center (CSMC) and a geographically distinct cohort of 5549 studies from Stanford Healthcare (SHC).

In the present study, a deep learning pipeline was developed and evaluated to detect and assess TR severity from transthoracic echocardiogram studies. Automating the entire process of view selection, identification of TR by color Doppler, and assessment of severity, it was hypothesized that a deep learning approach could assess TR severity with high-throughput automation. This pipeline was evaluated with data from 2 geographically distinct sites, including a temporally distinct test cohort separate from the training and validation cohorts (Figure 1). Combined with other echocardiography AI algorithms, like those enabling novices to obtain point of care echocardiographic images,^21^ such an approach could be used for serial surveillance and screening of TR.

## Methods

### Study Population and Data Source

#### Cedars-Sinai Medical Center Cohort

TTE studies at Cedars-Sinai Medical Center (CSMC) between October 4, 2011, and December 31, 2021, were used to train the deep learning pipeline (EchoNet-TR) for high-throughput TR identification and grading. Studies were initially sourced from Digital Imaging and Communications in Medicine (DICOM) files and underwent deidentification, view classification, and preprocessing into AVI files, as previously described,^25^ yielding 2 079 898 videos from 47 312 studies involving 31 708 patients. From these videos, 57 701 apical 4-chamber (A4C) videos with color Doppler across the tricuspid valve were manually curated and used to train a deep learning pipeline for TR phenotyping.

Studies were randomly split on a patient level into training (95%) and internal validation (5%) cohorts to train deep neural networks for TR phenotyping.^27^ Identical patient-level splits were maintained training both the TR severity and view classification models. The trained models were evaluated serially as a single pipeline on a held-out temporal test set of 2462 TTE studies (101 455 videos) from 2170 patients receiving care at CSMC between January 1, 2022, and June 4, 2022. Patients in the training and validation sets were excluded from the training set.

#### Stanford Healthcare Cohort

The pipeline was evaluated on 5549 studies (containing a total of 278 377 videos) from Stanford Healthcare (SHC)’s high-volume echocardiography lab. The automated view classification pipeline was compared with manual curation of videos within those studies to evaluate specificity. All videos identified by the view classifier were used for downstream TR severity model validation. Model output was compared with TR severity determined by expert cardiologists from the clinical reports. This study was approved by the institutional review boards at CSMC and SHC. Informed consent was waived, as the study involved secondary analysis of existing data without patient contact. Reporting of study results is consistent with guidelines put forth by Consolidated Standards of Reporting Trials-Artificial Intelligence (CONSORT-AI) reporting guidelines (eTable 12 in Supplement 1).^28,29^

### AI Model Training

The model pipeline consisted of a view classifier capable of detecting A4C videos with color Doppler across the tricuspid valve from full echocardiographic studies and a TR severity classification model. The PyTorch Lightning deep learning framework was used to train deep learning models. Video-based convolutional neural networks of the R(2 + 1)D architecture were used for view classification and TR severity assessment.^30^ The view classification model was initialized with random weights, while the TR severity model was initialized with weights from EchoNet-Dynamic.^27^ Both models were trained using a cross-entropy loss function for up to 100 epochs, an Adam optimizer, an initial learning rate of 1e-2, and a batch size of 24 on an NVIDIA GeForce RTX 3090 graphics processing unit (NVIDIA Corporation). Early stopping was performed based on the validation loss.

The view classifier was trained using the 57 701 manually curated A4C videos with color Doppler across the tricuspid valve as cases and 421 679 controls from the same studies. Controls consisted of any videos that were not A4C videos with color Doppler across the tricuspid valve and included videos of both A4C and other echocardiographic views (both with and without color Doppler information). The TR severity model was trained using 57 701 manually curated videos, which consisted of 14 318 videos with no TR, 16 507 videos with mild TR, 19 820 videos with moderate TR, and 7056 videos with severe TR (Figure 1). TR severity for each study was determined based on the clinical echocardiographic reports from CSMC’s high-volume echocardiography lab, where severity was assessed in accordance with American Society of Echocardiography guidelines.^31^ When TR was characterized as an intermediate category (ie, trace to mild or mild to moderate or moderate to severe), videos were placed in the more severe category. Studies with concomitant tricuspid stenosis, prosthetic valves, and heart failure were also included in both training and validation datasets.

### Statistical Analysis

The pipeline was then evaluated on 2 test sets not seen during model training: 2462 studies obtained at CSMC in 2022 (temporally distinct from the training and validation studies and with no patient overlap) and 5549 studies obtained at SHC in 2018. This process is summarized in Figure 1. Confusion matrices and area under the receiver operating characteristic curve (AUC) were used to assess model performance, and statistics related to TR model performance were calculated on a study level. When a study had more than 1 A4C video with Doppler information across the tricuspid valve, predictions across videos were ensembled, and the video that resulted in the greatest predicted TR severity was used for analysis. When multiple videos resulted in the same maximal predicted severity, the video with the highest prediction probability for the given level of TR severity was used. In both the internal and external test sets, AUC, F1 score, recall (sensitivity), positive predictive value (PPV), and negative predictive value (NPV) were calculated for clinically significant TR, which was defined as greater than moderate TR and severe TR. AUC was also calculated for relevant subsets in the CSMC test cohort. Statistical analysis was performed in Python version 3.8.0 (Python Software Foundation). Confidence intervals were computed via bootstrapping with 10 000 samples.

Subgroup analysis was conducted to assess model performance in patients with different ranges of right and LVEF, pulmonary artery pressure, associated comorbidities (≥mild right atrial dilation, ≥mild mitral regurgitation, ≥moderate aortic stenosis, and ≥moderate aortic regurgitation), study characteristics, and other clinical characteristics. Echocardiogram study quality was determined by clinicians and extracted from the clinical report. Studies where clinicians commented on technical difficulty, poor study quality, or where 1 or more major cardiac structure (left ventricle, right ventricle, pulmonary artery, etc) were not well visualized were classified as technically difficult.

### Error Mode Analysis

TTEs that were overclassified or underclassified by 1 class were evaluated, as these made up most model errors. Underclassified TTEs were evaluated to gauge if free-text TTE reports mentioned intermediate categories of TR (ie, mild to moderate or moderate to severe). Meanwhile, overclassified TTEs where right ventricular systolic pressure (RVSP) and right atrial or right ventricular (RA/RV) pressure gradient were available were compared to those that were correctly classified for differences in disease severity to better understand erroneous classification of the TR severity model. Continuous variables were compared using a Mann-Whitney *U* test to assess for differences in means.

### Model Explainability

Features identified by the TR severity model were evaluated using saliency mapping, generated using the Integrated Gradients method.^32^ This method generated a heatmap for every frame of the video, summarized as a final 2-dimensional heatmap generated by using the maximum value along the temporal axis for each pixel location in the video. Pixels brighter in intensity and closer to yellow were more salient to model predictions, while those darker in color were less important to the model’s final prediction. When assessing videos with no TR, heatmaps were obtained by taking the maximum of saliency maps for the moderate and severe class output neurons for each pixel location.

#### Comparison With Cardiac Magnetic Resonance Imaging

Concordance between model predictions, TTE labels, and magnetic resonance imaging (MRI) labels was evaluated. To accomplish this, a held-out set of 572 CSMC patients who did not have studies in the training or validation sets was identified. These patients received a total of at least 1 echocardiogram study within 180 days of cardiac MRI assessment of TR. When a patient had more than 1 TTE within 180 days of cardac MRI, the TTE that was obtained closest in time to the MRI was used for analysis. MRI labels were abstracted from clinical reports, which used quantitative evaluation to stratify TR at high levels and qualitative evaluation at lower levels.

## Results

### Study Population

Study and patient characteristics for TTEs used to train the view classification and TR severity models are shown in Table 1 and eTable 1 in Supplement 1, where statistics were calculated on a study level. In the CSMC training, validation, and test sets, patients had similar characteristics. Meanwhile, the CSMC and SHC test cohorts differed. The SHC test cohort contained lower numbers of studies from patients with moderate TR (5.0% vs 25.0%) and severe TR (4.4% vs 10.0%). Compared with the CSMC cohort, the SHC cohort also had a lower proportion of videos from Black patients (4.5% vs 14.5%) and a higher proportion of videos from Asian patients (25.2% vs 9.8%).

**Table 1.** Model Derivation Cohort and Test Cohort Characteristics

Characteristic	Studies, No. (%)			
	Derivation cohort		Test cohort	
	Training (n = 44 908)	Validation (n = 2404)	CSMC (n = 2462)	SHC (n = 5549)
Patients	30 125 (88.92)	1583 (4.67)	2170 (6.41)	5014 (NA)^b^
View classification model videos, No.	453 787	24 484	101 415	278 377
TR severity model videos, No.	54 787	3080	2914	7233
Sex				
Female	20 912 (47.0)	1110 (46.2)	1128 (47.1)^c^	945 (47.2)
Male	23 552 (53.0)	1282 (53.3)	1286 (52.2)	1057 (52.7)^a^
Hypertension	27 743 (61.8)	1477 (61.4)	1295 (52.6)	953 (47.6)^a^
Coronary artery disease	18 983 (42.3)	999 (41.6)	903 (36.7)	721 (36.0)^a^
Atrial fibrillation	14 612 (32.5)	856 (35.6)	515 (20.9)	550 (27.4)^a^
Ejection fraction, mean (SD), %	56.9 (15.4)	57.0 (15.1)	57.2 (15.1)	57.4 (11.0)^a^
Left atrial volume index, mean (SD), mL/m^2^	35.5 (15.7)	36.1 (16.0)	31.8 (15.5)	Not available
TR severity				
Control	12 297 (27.4)	627 (26.1)	829 (33.7)	3223 (58.1)
Mild	13 286 (29.6)	710 (29.5)	770 (31.3)	1808 (32.6)
Moderate	14 363 (32.0)	785 (32.7)	617 (25.1)	275 (5.0)
Severe	4962 (11.0)	282 (11.7)	246 (10.0)	243 (4.4)
Race^d^				
Asian	3459 (7.7)	192 (8.0)	241 (9.8)	311 (15.5)^a^
Black	6004 (13.4)	288 (12.0)	358 (14.5)	90 (4.5)^a^
White	31 117 (69.3)	1698 (70.6)	1559 (63.3)	1097 (54.8)^a^
Other^e^	4328 (9.6)	226 (9.4)	304 (12.3)	311 (15.5)^a^

Abbreviations: CSMC, Cedars-Sinai Medical Center; NA, not applicable; SHC, Stanford Healthcare; TR, tricuspid regurgitation.

^a^ A total of 2003 studies in the SHC test cohort had information on comorbidities, demographics, and ejection fraction.

^b^ The SHC cohort was only used for external validation.

^c^ Two studies across 2 patients in the test set had unknown sex.

^d^ Race was drawn from the electronic health record.

^e^ Other includes individuals identified as American Indian or Alaska Native, Native Hawaiian or Pacific Islander or whose race was unknown or other than those listed.

### View Classifier Performance Across 2 Institutions

On a test set of 2462 TTEs (101 415 videos) from CSMC not seen during model training, the view classifier had an AUC of 1.000 (95% CI, 0.999-1.000) and at a threshold of 0.800 and identified an A4C video with color doppler across the tricuspid valve in 2410 studies in the test set with a sensitivity of 0.979 (95% CI, 0.973-0.985) and a specificity of 1.000 (95% CI, 1.000-1.000). To evaluate generalization of the view classification model at a geographically distinct site, its performance was evaluated on 5549 studies (278 377 videos) from SHC. In this external validation set, the view classifier picked up at least 1 A4C tricuspid Doppler video in 5268 studies for a sensitivity of 0.949 (95% CI, 0.944-0.955) and a specificity of 1.000 (95% CI, 0.999-1.000).

### TR Severity Model Performance Across 2 Institutions

**Figure 2.**
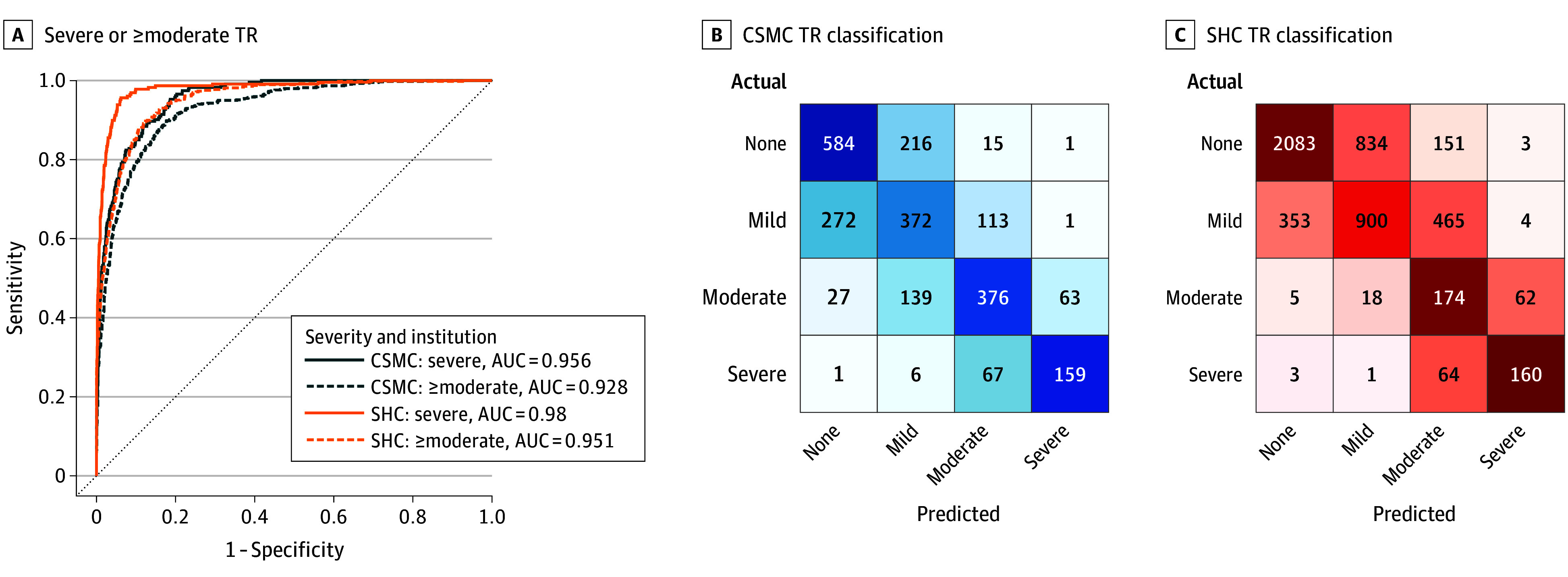
Model Performance Across Severity and Institution A, Receiver operating characteristic curves for detection of severe or at least moderate tricuspid regurgitation (TR) at Cedars-Sinai Medical Center (CSMC) and Stanford Healthcare (SHC). The at least moderate category (≥moderate) included moderate, moderate to severe, and severe TR. TR classification is shown on test set videos from CSMC (B) and SHC (C). Confusion matrix colormap values were scaled based on the proportion of actual disease cases in each class that were predicted in each possible disease category. This was done to allow for relative comparison of model performance across disease classes (none, mild, moderate, and severe), given class imbalance. Statistics and confusion matrices are reported on a study level. When a study had multiple TR Doppler videos, the video with the maximum predicted TR severity was used. When multiple videos lead to the same maximum severity model prediction, the video with the highest prediction probability for that severity of TR was used. AUC indicates area under the receiver operating characteristic curve.

The TR severity model showed strong performance in TR detection (Figure 2). In the temporally distinct CSMC test set, the model detected at least moderate TR (defined as moderate or severe TR) with an AUC of 0.928 (95% CI, 0.913-0.943) and detected severe TR with an AUC of 0.956 (95% CI, 0.940-0.969). Severe TR was ruled out with an NPV of 0.966 (95% CI, 0.955-0.977) and at least moderate TR was excluded with an NPV of 0.893 (95% CI, 0.871-0.914). Further information on TR model performance is presented in Table 2 and eTable 2 in Supplement 1. Strong model performance was preserved across institutions. In the 2018 SHC cohort, the model identified severe TR with an AUC of 0.980 (95% CI, 0.966-0.989) and at least moderate TR (defined as moderate or severe TR) with an AUC of 0.951 (95% CI, 0.938-0.962). In this cohort, the model demonstrated an NPV of 0.987 (95% CI, 0.982-0.991) for severe TR and an NPV of 0.994 (95% CI, 0.990-0.997) for at least moderate TR. Weighted Cohen κ, accuracy, and accuracy for different classes of clinically significant TR are shown in eTable 2 in Supplement 1. Similar results were yielded when the latest TTE available for each patient in the test cohort was used, as reported in eTables 3 4 in Supplement 1.

**Table 2.** Model Performance

Site or class	Value (95% CI)					
	AUC	PPV	NPV	Recall	Specificity	F1 score
**CSMC**						
≥Moderate TR	0.928 (0.913-0.943)	0.836 (0.799-0.871)	0.893 (0.871-0.914)	0.794 (0.753-0.831)	0.917 (0.904-0.931)	0.814 (0.784-0.843)
Severe TR	0.956 (0.940-0.969)	0.710 (0.622-0.791)	0.966 (0.955-0.977)	0.682 (0.596-0.765)	0.970 (0.963-0.977)	0.696 (0.622-0.759)
**SHC**						
≥Moderate TR	0.951 (0.938-0.962)	0.427 (0.383-0.466)	0.994 (0.990-0.997)	0.945 (0.914-0.971)	0.870 (0.860-0.880)	0.586 (0.544-0.626)
Severe TR	0.980 (0.966-0.989)	0.699 (0.614-0.780)	0.987 (0.982-0.991)	0.702 (0.616-0.784)	0.986 (0.983-0.990)	0.700 (0.630-0.763)

Abbreviations: AUC, area under the receiver operating characteristic curve; CSMC, Cedars-Sinai Medical Center; NPV, negative predictive value; PPV, positive predictive value; SHC, Stanford Healthcare; TR, tricuspid regurgitation.

### Subset Analysis

The TR severity model showed strong performance across test set subgroups (Table 3). In studies with normal, mildly depressed, or moderately or severely depressed right ventricular function, moderate or severe TR was detected with AUCs of 0.923 (95% CI, 0.902-0.942), 0.904 (95% CI, 0.847-0.951), and 0.861 (95% CI, 0.848-0.952), respectively. Meanwhile, severe TR was identified with AUCs of 0.962 (95% CI, 0.940-0.979), 0.924 (95% CI, 0.872-0.966), and 0.882 (95% CI, 0.873-0.966), respectively. Performance was similar across different LVEF ranges. The model also performed similarly well in studies from patients with a history of preexisting atrial fibrillation, RA dilation, and coexisting left-sided valvular heart disease, with moderate or severe TR detection ranging from 0.886 (95% CI, 0.804-0.954) to 0.918 (95% CI, 0.848-0.973) and severe TR detection ranging from 0.917 (95% CI, 0.975-0.952) to 0.961 (95% CI, 0.908-0.997).

**Table 3.** Tricuspid Regurgitation (TR) Model Subset Analysis

Subset	Studies, No.	TR severity model performance, AUC (95% CI)	
		Moderate/severe	Severe
Right ventricular function			
Normal function	1845	0.923 (0.902-0.942)	0.962 (0.940-0.979)
Mildly depressed	285	0.904 (0.847-0.951)	0.924 (0.872-0.966)
Moderately or severely depressed	266	0.861 (0.848-0.952)	0.882 (0.873-0.966)
Left ventricular systolic function			
LVEF ≥50%	1924	0.930 (0.913-0.947)	0.962 (0.944-0.977)
LVEF <50%	436	0.897 (0.851-0.937)	0.920 (0.876-0.957)
LVEF ≥35%	2105	0.932 (0.915-0.947)	0.960 (0.943-0.974)
LVEF <35%	255	0.860 (0.787-0.923)	0.917 (0.857-0.964)
Atrial fibrillation	499	0.908 (0.869-0.941)	0.917 (0.875-0.952)
Right atrial dilation	505	0.892 (0.847-0.931)	0.920 (0.882-0.952)
Pulmonary artery (PA) pressure			
PA pressure >25 mm Hg	1445	0.892 (0.871-0.916)	0.929 (0.905-0.950)
PA pressure ≤25 mm Hg	622	0.920 (0.835-0.982)	0.998 (0.990-1.000)
PA pressure >35 mm Hg	822	0.850 (0.806-0.890)	0.881 (0.841-0.916)
PA pressure ≤35 mm Hg	1245	0.915 (0.880-0.945)	0.990 (0.975-0.999)
Mitral regurgitation	357	0.896 (0.869-0.922)	0.945 (0.920-0.966)
Aortic stenosis	155	0.918 (0.848-0.973)	0.959 (0.901-0.996)
Aortic regurgitation	144	0.886 (0.804-0.954)	0.961 (0.908-0.997)
Study quality	253	0.927 (0.826-0.989)	0.982 (0.932-1.000)

Abbreviation: LVEF, left ventricular ejection fraction.

### MRI Comparison

Model predictions of TR severity were compared with assessments using cardiac magnetic resonance (CMR) imaging, another imaging modality capable of assessing valvular function. The MRI comparison cohort was composed of 572 studies from 572 patients. The cohort consisted of 359 studies (62.7%) with no TR, 187 studies (32.7%) with mild TR, 17 studies (2.9%) with moderate TR, and 9 studies (1.1%) with severe TR (eTable 5 in Supplement 1). Model-predicted TR severity showed strong concordance with CMR assessment of TR severity for moderate or severe TR (AUC: 0.896 [95% CI, 0.822-0.948]) and severe TR (AUC: 0.949 [95% CI, 0.845-0.999]; eTable 6 in Supplement 1). Similarly, cardiologist-determined TR severity from echocardiogram studies also agreed with cardiologist-determined severity using MRI for moderate or severe TR (0.820 [95% CI, 0.686-0.966]) and severe TR (0.841 [95% CI, 0.480-0.997]; eTable 7 in Supplement 1). Meanwhile, the difference in AUC between AI model predictions and cardiologist-based echocardiogram prediction vs MRI were significantly different for at least moderate TR (DeLong test, 0.885 vs 0.814; *P* = .02) but not severe TR (DeLong test, 0.849 vs 0.948; *P* = .13; eTable 8 in Supplement 1). Full results are shown in eFigure 1 and eTables 5-8 in Supplement 1.

### Error Mode Analysis

Moderate and severe TTEs underclassified by 1 class were most often mild to moderate (84.17%) or moderate to severe (68.66%; eTable 9 in Supplement 1). Meanwhile, only 2.2% of underclassified TTEs were noted as “Trace/trivial to Mild.” Overclassified TTEs (eTable 10 in Supplement 1) with documented RA/RV pressure gradients showed significantly increased mean (SD) RA/RV pressure gradients compared to their correctly classified counterparts for no TR (21.75 [8.72] vs 19.44 [13.44]; *P* < .01) and mild TR (30.35 [9.10] vs 26.96 [14.36]; *P* < .001). Similar results were seen for mean (SD) RVSP for no TR (27.08 [8.55] vs 21.80 [6.02]; *P* < .001), mild TR (34.98 [10.23] vs 30.93 [9.88]; *P* = .02), and moderate TR (50.43 [17.59] vs 46.12 [28.43]; *P* = .03) cases that were overclassified. Distributions and means of each set are shown in eFigure 2 in Supplement 1.

### Model Explainability

The Integrated Gradients method was used to create saliency maps that identified regions of interest in each video contributing the most to detection of TR severity (eFigure 3 in Supplement 1).^32^ Saliency maps for the TR severity model demonstrated that the clinically relevant imaging features of TR were important for model predictions, with activation signal localizing to pixels in the color Doppler window and primarily highlighting the TR jet, indicating that the model used appropriate physiologic features of TR to make predictions.

## Discussion

The current work presents a comprehensive, automated pipeline capable of characterizing TR from full echocardiogram studies. In a temporally distinct test cohort, the AI automation pipeline demonstrated strong performance in identifying cases of severe and at least moderate TR, with an AUC greater than 0.928 and an NPV greater than 0.89. The EchoNet-TR algorithm generalized to thousands of studies from a large, geographically distinct cohort with similar performance. While further work remains, these results present a deep learning model that could aid in screening for clinically significant TR. With open-source code and weights, this project can serve as the foundation for future prospective evaluation of AI-assisted workflows in echocardiography.

In AI applied to echocardiography, the right heart is still underrepresented. While prior work has shown AI’s ability to characterize LVEF, left ventricular hypertrophy, and left-sided valvular lesions,^10,19,32,33^ there has been little work on TR. The present work applies a state-of-the-art video-based architecture for TR detection on a large training dataset and shows that such an approach trains a generalizable AI model for the assessment of regurgitant severity. Despite relying on a single salient view, EchoNet-TR reliably matches expert cardiologists’ assessments. Despite such limited information, the performance is comparable to clinical variation across clinicians and across modalities. The current pipeline focusing on the A4C view alone suggests there is richness of ancillary information (RA enlargement, RV systolic function, etc) that augments the assessment of TR severity, even without 3-D information. This potential use of information mimics clinical decision-making, wherein cardiologists often integrate conflicting metrics from different views and ancillary information to arrive at a final TR severity.

### Limitations

There are a few limitations worth considering. While PPV for at least moderate TR was lower in the SHC cohort, the lower population prevalence of such cases (10% vs 35%) was the likely driver, as specificity (0.870 [95% CI, 0.860-0.880]) was preserved. Similarly, despite weighted Cohen κ and exact accuracy being lower than other metrics of model performance, at least moderate TR and severe TR were still detected with accuracy of more than 0.824 (95% CI, 0.802-0.846) in both test cohorts, indicating that higher misclassification rates of mild TR and no TR were responsible for the lower exact accuracy. Additionally, EchoNet-TR was trained on study-level clinician assessments of TR severity. TR evaluation is subject to interobserver variability, and although the large scale of data used here could attenuate some of this variability, training and evaluating models on these labels have the inherent risk of propagating this variability, especially as the large scale of data in this study did not allow for manual adjudication prior to model training. Future work can focus on more nuanced evaluation of disease, like automatic quantification of regurgitant volume, valve leaflet thickness, orifice area, etc.

A few notable findings with regard to RV function stand out in reviewing cases misclassified by this study’s model. Studies that were overclassified by 1 level of severity showed a statistically significant difference in RA/RV pressure gradient and RVSP than those correctly classified, possibly suggesting more severe disease and highlighting possible subtle nuances in disease severity detected by the algorithm. This is also important when considering the algorithm’s limitation in differentiating between low levels of TR (no TR and mild TR). Similarly, in the internal test set, most underclassification errors in moderate and severe studies occurred in studies that belonged to intermediate levels of severity, reflecting possible limitation in the use of 4 severity categories or interobserver variability during classification (eTable 9 in Supplement 1).

Finally, while echocardiography remains first-line, multimodal TR characterization is of interest. The algorithm’s decreased performance on CMR-based labels (eTables 5-8 in Supplement 1) highlights the imperfect multimodal concordance, even by expert readers.^34,35^ However, the authors caution against the notion that CMR should be the criterion standard in assessing severity of TR. Further research on TR characterization across modalities is required, however, recognizing that CMR has lower temporal resolution,^6^ greater slice thickness,^6^ requires labor intensive segmentation, and relies on software that is newer, proprietary, and less validated.^36^

## Conclusions

In summary, we introduce EchoNet-TR, a model for TR screening from single-view TTE videos with excellent performance and generalizability. In this study, we provide a workflow for isolating tricuspid valve color Doppler videos and assessing TR severity with strong AUC in 2 distinct test cohorts. While more work remains, the current algorithm could potentially be used to increase access to TR screening or enable retrospective institutional database review.^21,37^

## Notes

Supplement 1. eTable 1. TR Severity Model Derivation Cohort and Test Cohort Characteristics eTable 2. Cohen’s Kappa and Accuracy Analysis eTable 3. Patient Level Model Performance eTable 4. Patient Level Analysis of Accuracy & Cohen’s Kappa eTable 5. MRI Cohort Characteristics eTable 6. Echocardiogram Severity vs MRI Severity eTable 7. Model Predicted Severity vs MRI Severity eTable 8. DeLong Test Results eTable 9. Underclassification Error Mode Analysis eTable 10. Overclassified Cases - RA/RV Gradient eTable 11. Overclassified Cases - RVSP eTable 12. CONSORT-AI Checklist eFigure 1. Model Predictions, MRI Labels, and TTE Labels eFigure 2. Error Mode Analysis of Overclassified Cases eFigure 3. Saliency Map Visualization for TR Classification Models

Supplement 2. Data Sharing Statement
