# Supplementary material for: Automated Deep Learning Phenotyping of Tricuspid Regurgitation in Echocardiography
Source: JAMA Cardiol. 2025 Apr 16;10(6):595–602. doi: 10.1001/jamacardio.2025.0498 (PMC12004246; doi:10.1001/jamacardio.2025.0498)
Supplement: Supplement 1. — eTable 1. TR Severity Model Derivation Cohort and Test Cohort Characteristics eTable 2. Cohen’s Kappa and Accuracy Analysis eTable 3. Patient Level Model Performance eTable 4. Patient Level Analysis of Accuracy & Cohen’s Kappa eTable 5. MRI Cohort Characteristics eTable 6. Echocardiogram Severity vs MRI Severity eTable 7. Model Predicted Severity vs MRI Severity eTable 8. DeLong Test Results eTable 9. Underclassification Error Mode Analysis eTable 10. Overclassified Cases - RA/RV Gradient eTable 11. Overclassified Cases - RVSP eTable 12. CONSORT-AI Checklist eFigure 1. Model Predictions, MRI Labels, and TTE Labels eFigure 2. Error Mode Analysis of Overclassified Cases eFigure 3. Saliency Map Visualization for TR Classification Models [file jamacardiol-e250498-s001.pdf]

Supplemental Online Content

Vrudhula A, Vukadinovic M, Haeffele C, et al. Automated deep learning phenotyping of tricuspid regurgitation in echocardiography. *JAMA Cardiol*. Published online April 9, 2025. doi:10.1001/jamacardio.2025.0498

eTable 1. TR Severity Model Derivation Cohort and Test Cohort Characteristics
eTable 2. Cohen's Kappa and Accuracy Analysis
eTable 3. Patient Level Model Performance
eTable 4. Patient Level Analysis of Accuracy & Cohen's Kappa
eTable 5. MRI Cohort Characteristics
eTable 6. Echocardiogram Severity vs MRI Severity
eTable 7. Model Predicted Severity vs MRI Severity
eTable 8. DeLong Test Results
eTable 9. Underclassification Error Mode Analysis
eTable 10. Overclassified Cases - RA/RV Gradient
eTable 11. Overclassified Cases - RVSP
eTable 12. CONSORT-AI Checklist
eFigure 1. Model Predictions, MRI Labels, and TTE Labels
eFigure 2. Error Mode Analysis of Overclassified Cases
eFigure 3. Saliency Map Visualization for TR Classification Models

This supplemental material has been provided by the authors to give readers additional information about their work.

© 2025 Vrudhula A et al. *JAMA Cardiology*.

eTable 1. TR Severity Model Derivation Cohort and Test Cohort Characteristics

	Derivation Cohorts		Test Cohorts	
	**Train**	**Val**	**CSMC**	**SHC**
**Patients**	30,125 (88.92)	1,583 (4.67)	2,132 (6.41)	4,798
**Studies**	44,908	2,404	2,412	5,280
**Videos**	54,787	3,080	2,914	7,233
**Male**	23,552 (53.0)	1,282 ()	1263 (52.3)	1,057 (52.7) *
**Hypertension**	27,743 (61.8)	1,477 (61.4)	1271 (52.7)	898 (47.3) *
**Coronary artery disease**	18,983 (42.3)	999 (41.6)	884 (36.7)	674 (35.5) *
**Atrial fibrillation**	14,612 (32.5)	856 (35.6)	499 (20.7)	520 (27.4) *
**Ejection fraction (EF)**	56.9 (15.4)	57.0 (15.1)	57.2 (16.9)	57.5 (10.8)*
EF ≥ 35	39,294 (89.5)	2,111 (90.0)	2105 (89.1)	1,796 (93.7) *
EF < 35	4,616 (10.5)	236 (10.0)	255 (10.8)	120 (6.3) *
EF ≥ 50	35,287 (80.4)	1,889 (80.5)	1924 (81.5)	1,585 (82.7) *
EF < 50	8623 (19.6)	458 (19.5)	436 (18.5)	331 (17.2) *
**Left atrial volume index**	35.5 (15.7)	36.1 (16.0)	31.8 (15.3)	*Not assessed*
**TR severity**				
**Control**	12297 (27.4)	627 (26.1)	816 (33.8)	3,071 (58.2)
**Mild**	13286 (29.6)	710 (29.5)	758 (31.4)	1,722 (32.6)
**Moderate**	14363 (32.0)	785 (32.7)	605 (25.1)	259 (4.91)
**Severe**	4962 (11.0)	282 (11.7)	233 (9.7)	228 (4.32)
**Race**				
**White**	31,117 (69.3)	1,698 (70.6)	1,559 (63.3)	1,097 (54.8)
**Black**	6,004 (13.4)	288 (12.0)	358 (14.5)	90 (4.5)
**Asian**	3,459 (7.7)	192 (8.0)	241 (9.8)	504 (25.2)
**Other**	4,328 (9.6)	226 (9.4)	304 (12.3)	311 (15.5)
**Right ventricular dysfunction**				
Mildly depressed	7,433 (13.6)	374 (12.8)	415 (12.3)	*Not assessed*
Moderately/severely depressed	6,027 (11.0)	301 (10.3)	409 (12.1)	
**Pulmonary artery (PA) pressure**				
PA pressure > 35 mm Hg	17,646 (44.6)	976 (46.1)	822 (39.7)	*Not assessed*
PA pressure ≤ 35 mm Hg	21,866 (55.4)	1,140 (53.9)	1,245 (60.2)	
PA pressure > 25 mm Hg	29,638 (75.0)	1,616 (76.4)	1,445 (70.0)	
PA pressure ≤ 25 mm Hg	9,895 (25.0)	500 (23.6)	622 (30.0)	
**Mitral regurgitation**	26,412 (48.6)	1,438 (49.6)	1028 (42.6)	76 (4.0) *
**Aortic regurgitation**	4,602 (8.5)	232 (8.0)	144 (6.0)	29 (1.5) *
**Aortic stenosis**	3,668 (6.8)	218 (7.5)	155 (6.4)	209 (11.0) *
**Difficult study**	7,566 (13.8)	446 (15.3)	179 (7.4)	*Not assessed*

**1916 studies in the SHC test cohort had information on demographics and EF. 1897 studies in the SHC test cohort that had information on comorbidities.*

© 2025 Vrudhula A et al. *JAMA Cardiology*.

eTable 2. Cohen's Kappa and Accuracy Analysis

	Cohen's Kappa	Accuracy				
	**4 Class Severity**	**4 Class Severity**	**Accuracy within One Class**	**Moderate TR**	**≥ Moderate TR**	**Severe TR**
**CSMC**	0.768 (0.742 – 0.792)	0.618 (0.590 – 0.646)	0.979 (0.970-0.987)	0.824 (0.802 - 0.846)	0.874 (0.856 - 0.892)	0.942 (0.929 – 0.955)
**Stanford**	0.665 (0.636-0.691)	0.628 (0.609 - 0.647)	0.968 (0.962 – 0.975)	0.855 (0.842 - 0.869)	0.877 (0.864 – 0.889)	0.974 (0.968 – 0.980)

© 2025 Vrudhula A et al. *JAMA Cardiology*.

eTable 3. Patient Level Model Performance

Site	Class	AUROC	PPV	NPV	Recall	Specificity	F1-Score
CSMC	≥Moderate TR	0.929 (0.911 - 0.944)	0.818 (0.775 - 0.861)	0.905 (0.883-0.925)	0.779 (0.733 - 0.824)	0.924 (0.910 - 0.937)	0.798 (0.763 - 0.832)
	Severe TR	0.959 (0.941 - 0.974)	0.690 (0.583 - 0.790)	0.974 (0.964 - 0.984)	0.681 (0.580 - 0.782)	0.974 (0.968 - 0.982)	0.686 (0.599 - 0.762)
SHC	≥ Moderate TR	0.954 [0.941, 0.965]	0.953 (0.940 - 0.965)	0.427 (0.382 - 0.472)	0.995 (0.991 - 0.997)	0.951 (0.919 - 0.977)	0.876 (0.866 - 0.885)
	Severe TR	0.978 [0.963, 0.989]	0.978 (0.963 – 0.989)	0.685 (0.590 - 0.773)	0.987 (0.982 - 0.991)	0.692 (0.598 - 0.783)	0.986 (0.983-0.990)

© 2025 Vrudhula A et al. *JAMA Cardiology*.

eTable 4. Patient Level Analysis of Accuracy & Cohen's Kappa

	Cohen's Kappa	Accuracy				
	4 Class Severity	4 Class Severity	Accuracy within One Class	Moderate TR	≥ Moderate TR	Severe TR
**CSMC**	0.751 (0.720-0.779)	0.621 (0.593 - 0.650)	0.978 (0.969 - 0.987)	0.824 (0.802 - 0.846)	0.874 (0.856 - 0.892)	0.953 (0.94 - 0.965)
**Stanford**	0.662 (0.631 - 0.690)	0.632 (0.613 - 0.651)	0.969 (0.962 - 0.975)	0.860 (0.829 - 0.858)	0.882 (0.869 - 0.895)	0.958 (0.950-0.966)

© 2025 Vrudhula A et al. *JAMA Cardiology*.

eTable 5. MRI Cohort Characteristics

	**Count**
**Number of Patients**	572
**Number of Studies**	572
**TR Severity (Studies)**	
**None**	359 (62.7)
**Mild**	187 (32.7)
**Moderate**	17 (2.9)
**Severe**	9 (1.1)

eTable 6. Echocardiogram Severity vs MRI Severity

**Severity**	**AUC**
**Moderate/Severe**	0.814 (0.622 - 0.973)
**Severe**	0.849 (0.328 – 1.000)

eTable 7. Model Predicted Severity vs MRI Severity

**Severity**	**AUC**
**Moderate/Severe**	0.885 (0.777 - 0.970)
**Severe**	0.948 (0.751 - 1.000)

eTable 8. DeLong Test Results

**Severity**	**p-value**
**Moderate/Severe**	0.02
**Severe**	0.13

© 2025 Vrudhula A et al. *JAMA Cardiology*.

eTable 9. Underclassification Error Mode Analysis

TR Severity	Number Under classified by One Class	Number with Intermediate	Percent
**Mild**	272	6	2.21%
**Moderate**	139	117	84.17%
**Severe**	67	46	68.6%

© 2025 Vrudhula A et al. *JAMA Cardiology*.

eTable 10 - Overclassified Cases - RA/RV Gradient

	Correctly Classified			Overclassified by One Class			Mann Whitney U Test p-Value
Severity	No. Studies	No. Studies with Documented Gradient	Mean ± SD	No. Studies	No. Studies with Documented Gradient	Mean ± SD	
Control	584	285	19.44±13.44	216	118	21.75 ± 8.72	<0.01
Mild	372	307	26.96±14.36	113	95	30.35±9.10	<1e-4
Moderate	376	314	38.63±13.32	63	57	41.3±14.50	0.21

© 2025 Vrudhula A et al. *JAMA Cardiology*.

eTable 11. Overclassified Cases - RVSP

Abbreviation: RVSP, right ventricular systolic pressure

	Correctly Classified			Overclassified by One Class			Mann Whitney U Test p-Value
**Severity**	**No. of Studies**	**No. Studies with Documented RVSP**	**Mean ± SD**	**No. of Studies**	**No. Studies with Documented RVSP**	**Mean ± SD**	
Control	584	161	21.80±6.02	216	65	27.08±8.55	1e-5
Mild	372	152	30.93±9.88	113	52	34.98±10.23	0.02
Moderate	376	164	46.12±28.43	63	28	50.43±17.59	0.03

© 2025 Vrudhula A et al. *JAMA Cardiology*.

eTable 12. CONSORT-AI Checklist of Information to Include When Reporting a Randomized Trials of AI Interventions

Section	Item	CONSORT 2010 Item[a]	CONSORT-AI Item		Addressed on Page No[b]
Title and Abstract					
**Title and Abstract**	1a	Identification as a randomised trial in the title	CONSORT-AI 1a,b Elaboration	(i) Indicate that the intervention involves artificial intelligence/machine learning in the title and/or abstract and specify the type of model.	1
	1b	Structured summary of trial design, methods, results, and conclusions (for specific guidance see CONSORT for abstracts)		(ii) State the intended use of the AI intervention within the trial in the title and/or abstract.	1,2
Introduction					
**Background and objectives**	2a	Scientific background and explanation of rationale	CONSORT-AI 2a (i) Extension	Explain the intended use of the AI intervention in the context of the clinical pathway, including its purpose and its intended users (e.g. healthcare professionals, patients, public).	2,3
	2b	Specific objectives or hypotheses			2,3
Methods					
**Trial design**	3a	Description of trial design (such as parallel, factorial) including allocation ratio			n/a
	3b	Important changes to methods after trial commencement (such as eligibility criteria), with reasons			n/a
**Participants**	4a	Eligibility criteria for participants	CONSORT-AI 4a (i) Elaboration	State the inclusion and exclusion criteria at the level of participants.	4
			CONSORT-AI 4a (ii) Extension	State the inclusion and exclusion criteria at the level of the input data.	4 (reference 8)
	4b	Settings and locations where the data were collected	CONSORT-AI 4b Extension	Describe how the AI intervention was integrated into the trial setting, including any onsite or offsite requirements.	n/a
**Interventions**	5	The interventions for each group with sufficient details to allow replication, including how and when they were actually administered	CONSORT-AI 5 (i) Extension	State which version of the AI algorithm was used.	5
			CONSORT-AI 5 (ii) Extension	Describe how the input data were acquired and selected for the AI intervention.	4 (reference 8)
			CONSORT-AI 5 (iii) Extension	Describe how poor quality or unavailable input data were assessed and handled.	4 (reference 8)
			CONSORT-AI 5 (iv) Extension.	Specify whether there was human-AI interaction in the handling of the input data, and what level of expertise was required of users.	n/a
			CONSORT-AI 5 (v) Extension	Specify the output of the AI intervention	5
			CONSORT-AI 5 (vi) Extension	Explain how the AI intervention's outputs contributed to decision-making or other elements of clinical practice.	n/a
**Outcomes**	6a	Completely defined pre-specified primary and secondary outcome measures, including how and when they were assessed			5-6

© 2025 Vrudhula A et al. *JAMA Cardiology*.

	6b	Any changes to trial outcomes after the trial commenced, with reasons			n/a
**Sample size**	7a	How sample size was determined			4
	7b	When applicable, explanation of any interim analyses and stopping guidelines			n/a
Randomisation					
**Sequence generation**	8a	Method used to generate the random allocation sequence			n/a
	8b	Type of randomisation; details of any restriction (such as blocking and block size)			4
**Allocation concealment mechanism**	9	Mechanism used to implement the random allocation sequence (such as sequentially numbered containers), describing any steps taken to conceal the sequence until interventions were assigned			n/a
**Implementation**	10	Who generated the random allocation sequence, who enrolled participants, and who assigned participants to interventions			n/a
**Blinding**	11a	If done, who was blinded after assignment to interventions (for example, participants, care providers, those assessing outcomes) and how			n/a
	11b	If relevant, description of the similarity of interventions			n/a
**Statistical methods**	12a	Statistical methods used to compare groups for primary and secondary outcomes			5
	12b	Methods for additional analyses, such as subgroup analyses and adjusted analyses			5
Results					
**Participant flow** (a diagram is strongly recommended)	13a	For each group, the numbers of participants who were randomly assigned, received intended treatment, and were analysed for the primary outcome			Figure 2
	13b	For each group, losses and exclusions after randomisation, together with reasons			Figure 2
**Recruitment**	14a	Dates defining the periods of recruitment and follow-up			4
	14b	Why the trial ended or was stopped			n/a
**Baseline data**	15	A table showing baseline demographic and clinical characteristics for each group			15-16
**Numbers analysed**	16	For each group, number of participants (denominator) included in each analysis and whether the analysis was by original assigned groups			18
**Outcomes and estimation**	17a	For each primary and secondary outcome, results for each group, and the estimated effect size and its precision (such as 95% confidence interval)			18

© 2025 Vrudhula A et al. *JAMA Cardiology*.

	17b	For binary outcomes, presentation of both absolute and relative effect sizes is recommended			n/a
**Ancillary analyses**	18	Results of any other analyses performed, including subgroup analyses and adjusted analyses, distinguishing pre-specified from exploratory			Page 17, 18
**Harms**	19	All important harms or unintended effects in each group (for specific guidance see CONSORT for harms)	CONSORT-AI 19 Extension	Describe results of any analysis of performance errors and how errors were identified, where applicable. If no such analysis was planned or done, explain why not.	n/a
Discussion					
**Limitations**	20	Trial limitations, addressing sources of potential bias, imprecision, and, if relevant, multiplicity of analyses			9-10
**Generalisability**	21	Generalisability (external validity, applicability) of the trial findings			9-10
**Interpretation**	22	Interpretation consistent with results, balancing benefits and harms, and considering other relevant evidence			9-10
Other Information					
**Registration**	23	Registration number and name of trial registry			n/a
**Protocol**	24	Where the full trial protocol can be accessed, if available			n/a
**Funding**	25	Sources of funding and other support (such as supply of drugs), role of funders	CONSORT-AI 25 Extension.	State whether and how the AI intervention and/or its code can be accessed, including any restrictions to access or re-use.	11

[a] We strongly recommend reading this statement in conjunction with the CONSORT 2010 Explanation and Elaboration for important clarifications on all the items.
[b] Indicates page numbers to be completed by authors during protocol development.

© 2025 Vrudhula A et al. *JAMA Cardiology*.

eFigure 1. Model Predictions, MRI Labels, and TTE Labels

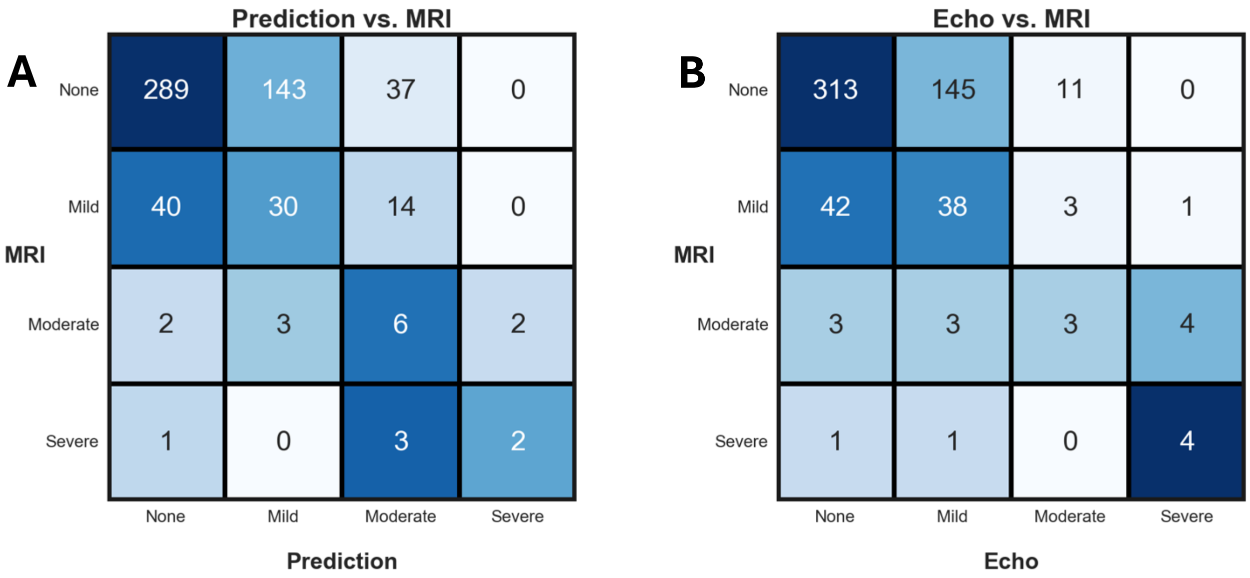

A. Confusion Matrix of Al Model Assessment of TR severity vs. MRI Assessment of TR severity
B. Confusion Matrix Cardiologist Echo-based Assessment of TR severity vs. MRI Assessment of TR severity

© 2025 Vrudhula A et al. *JAMA Cardiology*.

eFigure 2. Error Mode Analysis of Overclassified Cases

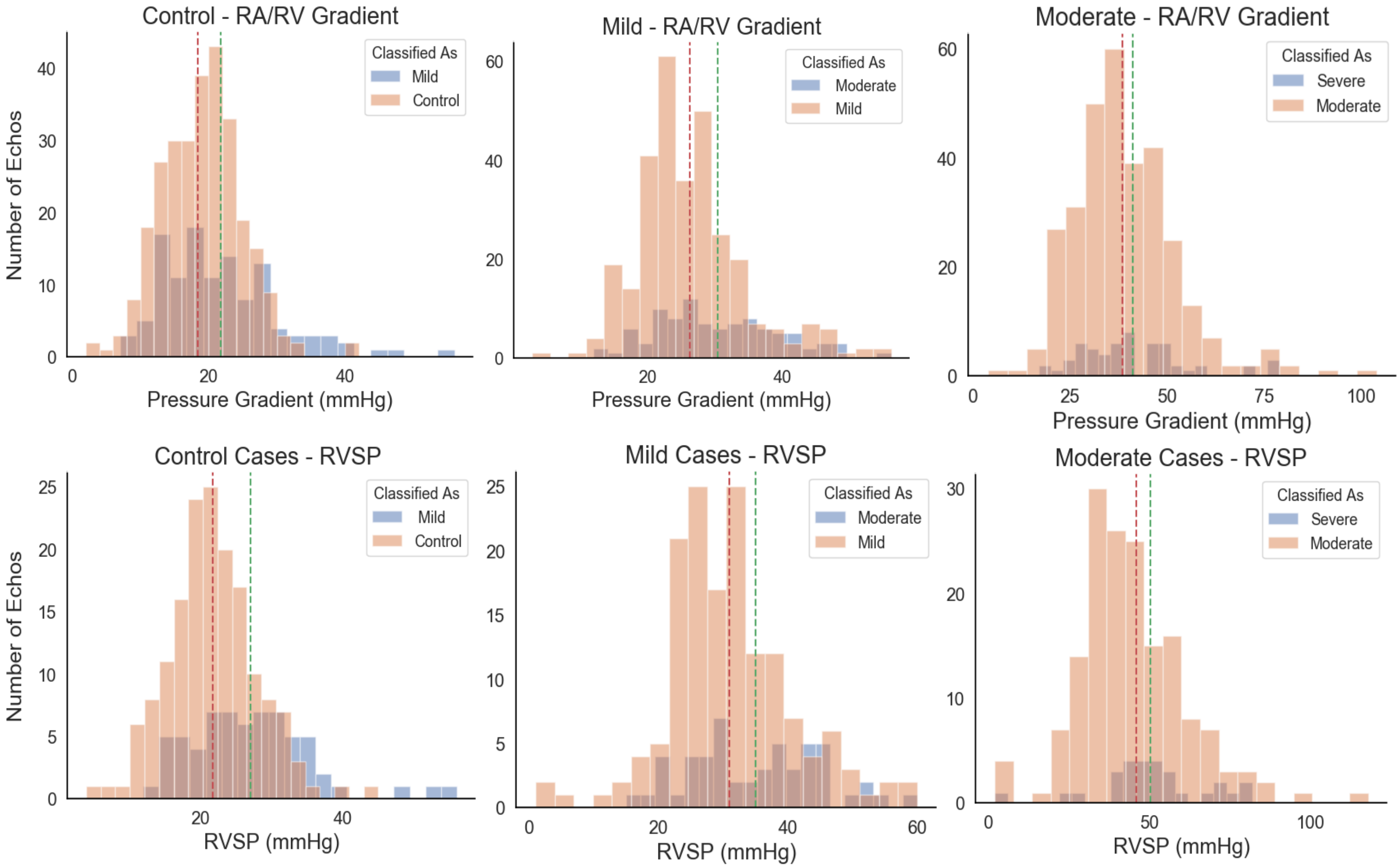

Distributions of RA/RV gradient (top row) and RVSP (bottom row) for correctly classified and overclassified TTEs. Dashed red and green lines represent the means of correctly classified and overclassified TTEs.

© 2025 Vrudhula A et al. *JAMA Cardiology*.

eFigure 3. Saliency Map Visualization for TR Classification Models

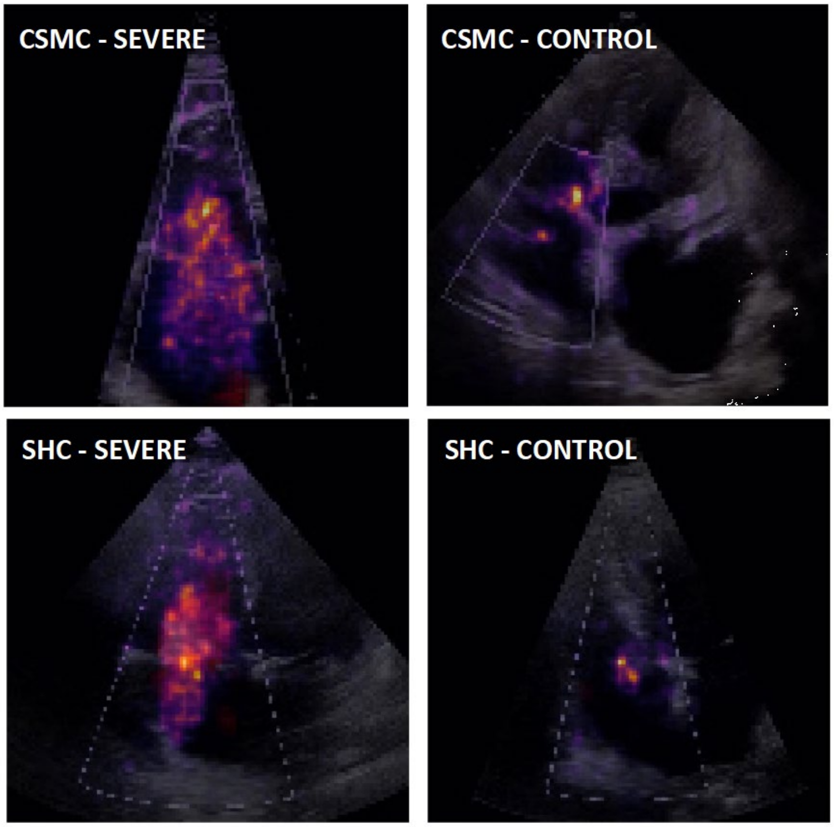

Videos with severe TR from CSMC (top left) and SHC (bottom left) and videos with no TR from CSMC (top right) and SHC (bottom right) are included. Saliency maps were computed using the Integrated Gradients method. The maximum value along the temporal axis for each pixel location was used to generate the final 2-dimensional heatmap. In the colormap, pixels more salient to model predictions are brighter in color and closer to yellow. Pixels darker in color are less influential to the model's final prediction. Severe TR videos were assessed by using the activation function for severe TR output neuron to generate a heatmap. When assessing controls (cases with no TR), heatmaps were generated by stacking heatmaps for severe and moderate TR output neurons and taking the maximum between the two at each pixel location.

© 2025 Vrudhula A et al. *JAMA Cardiology*.
