# Supplementary material for: Automated Deep Learning Phenotyping of Tricuspid Regurgitation in Echocardiography
Source: JAMA Cardiol. 2025 Apr 16;10(6):595–602. doi: 10.1001/jamacardio.2025.0498 (PMC12004246; doi:10.1001/jamacardio.2025.0498)
Supplement: Supplement 2. — Data Sharing Statement [file jamacardiol-e250498-s002.pdf]

Data Sharing Statement

Vrudhula. Automated Deep Learning Phenotyping of Tricuspid Regurgitation in Echocardiography. *JAMA Cardiol*. Published April 16, 2025. doi:10.1001/jamacardio.2025.0498

Data
Data available: No
